# Internally and externally generated emotions in people with acquired brain injury: preservation of emotional experience after right hemisphere lesions

**DOI:** 10.3389/fpsyg.2015.00101

**Published:** 2015-02-16

**Authors:** Christian E. Salas Riquelme, Darinka Radovic, Osvaldo Castro, Oliver H. Turnbull

**Affiliations:** ^1^Head Forward Rehabilitation CentreManchester, UK; ^2^School of Psychology, College of Health and Behavioral Sciences, Bangor UniversityBangor, UK; ^3^Manchester Institute of Education, Manchester UniversityManchester, UK; ^4^Escuela de Terapia Ocupacional, Facultad de Ciencias de la Salud, Universidad Autónoma de ChileSantiago, Chile

**Keywords:** emotion elicitation, emotional experience, brain injury, right hemisphere, emotion

## Abstract

The study of emotional changes after brain injury has contributed enormously to the understanding of the neural basis of emotion. However, little attention has been placed on the methods used to elicit emotional responses in people with brain damage. Of particular interest are subjects with right hemisphere [RH] cortical lesions, who have been described as presenting impairment in emotional processing. In this article, an internal and external mood induction procedure [MIP] was used to trigger positive and negative emotions, in a sample of 10 participants with RH damage, and 15 healthy controls. Emotional experience was registered by using a self-report questionnaire. As observed in previous studies, internal and external MIPs were equally effective in eliciting the target emotion, but the internal procedure generated higher levels of intensity. Remarkably, participants with RH lesions were equally able to experience both positive and negative affect. The results are discussed in relation to the role of the RH in the capacity to experience negative emotions.

## Introduction

Research on emotional changes after acquired brain injury has a long history (Gainotti, 1972, 2001; Borod, 2000; Adolphs, 2007), contributing to a growing understanding of the neural basis of several emotional processes, such as emotional *perception* (e.g., Adolphs et al., 2000; Borod et al., 2010; Tsuchida and Fellows, 2012), *expression* (e.g., Nakhutina et al., 2006; Kazandjian et al., 2007; Borod et al., 2010) and *experience* (e.g., Anderson and Phelps, 2002; Hornak et al., 2003; Gillihan et al., 2010; Feinstein et al., 2010a,b; Feinstein, 2013).

Typically, studies on emotion use some type of mood induction procedure [MIP] to trigger the intended target emotion. There are a wide range of MIP, such as self-referential statements (e.g., Velten, 1968; Kenealy, 1986), autobiographical recall (e.g., Brewer and Doughtie, 1980; Turnbull et al., 2004; Schaefer and Philippot, 2005), imagery (e.g., Wright and Mischel, 1982; Tranel et al., 1998) or film clips (e.g., Gross and Levenson, 1995; Rottenberg et al., 2007; Schaefer et al., 2010). However, there is little research comparing the effectiveness of each method (Isen and Gorgoglione, 1983; Gerrards-Hesse et al., 1994; Westermann et al., 1996). Recently, it has been reported that internal MIPs [autobiographical recall] generate higher levels of overall affect compared to external MIPs [film clips] (Salas et al., 2012). Because the successful triggering of target emotions is a prerequisite for measuring other more complex emotional processes, such as emotional comprehension or regulation (Rosen and Levenson, 2009), the comparative effectiveness of different forms of MIP is, itself, an important topic of research.

Notably, the correct selection of an MIP may well be critical when experimental subjects present with cognitive impairments that compromise the effective engagement with the stimuli, especially after acquired brain injury (Levenson, 2007). For example, patients might struggle recalling details of personal events [autobiographical recall], or grasping the plot on a film [film clips] (Levenson et al., 2008). Unfortunately, there has been no systematic research effort to address this issue. Thus, it seems timely to explore which MIPs are best suited to elicit emotional states in neurological patients.

Among the variety of patients with acquired brain damage, subjects with unilateral lesions to the right hemisphere (RH) are of particular interest for the topic of emotion elicitation. It has been widely reported that, compared to subjects with left unilateral lesions, they present a wide range of perceptual and expressive emotional deficits (for a review see Borod et al., 2010), as well as physiological hypo-reactivity to emotional stimuli (Heilman et al., 1978; Morrow et al., 1981). Furthermore, it has been suggested that RH lesions compromise specifically the processing of negative, or withdrawal, emotions (for a review Gainotti, 2000), often described as the *valence* hypothesis (Davidson, 1992a,b, 2001; Craig, 2005).

Unfortunately the evidence addressing emotion elicitation in neurological patients is modest and presents important methodological limitations. For example, most of the studies have focused on perceptual and expressive impairments, neglecting whether or not RH damage compromises the *subjective* experience of negative emotions. Additionally, the studies that have addressed changes in emotional intensity have used ratings by naïve judges as a method, thus not considering the patient's report of his own experience (Borod et al., 1996; Montreys and Borod, 1998). To our knowledge there are only two case studies that have experimentally addressed this problem, using an internal MIP [affective story recall] to explore RH patient's capacity to experience negative emotions (Turnbull et al., 2004; Tondowski et al., 2007). Both reported that individuals with RH lesions *were* capable of experiencing similar levels of negative emotions compared with controls, thus challenging the valence hypothesis. Unfortunately these studies are based on the report of a single case (Turnbull et al., 2004) or two cases (Tondowski et al., 2007), opening the question of generalization. In addition, a further limitation is that results are exclusively based on the rating of naïve judges, thus not considering the patients subjective emotional experience.

The present study is the first to offer some insight into this problem, by comparing the effectiveness of two different MIPs, in participants with unilateral RH damage, and a matched sample of healthy controls. Following previous work on emotion elicitation (Salas et al., 2012), internal [affective story recall] and external [film clip] MIPs were used to elicit positive [amusement] and negative [sadness] emotions. The affective experience of participants was registered using an adaptation of a well stablished self-report questionnaire (PANAS-X, Watson and Clark, 1994).

The present study thus attempts to extend the findings of Salas et al. (2012), which were based on a student sample, to the key population of patients with RH damage, and neurologically normal elderly controls. In addition, this study also attempts to extend the findings of Turnbull et al. (2004), this time using both internal and external MIPs, and self-reports, to test whether participants with RH were able to experience negative emotions [sadness]. Based on the results of Salas et al. (2012) the following predictions were considered: (1) *Selectivity*: That internal and external MIPs will selectively trigger the target emotion, but not the non-target emotions, in both healthy controls and neurological patients; (2) *Intensity*: That internal MIPs will generate significantly higher levels of self-reported emotion, compared with external MIPs, in both groups; (3) *Right Hemisphere*: that patients with RH lesions will report lower levels of negative emotional intensity than controls, when using both MIPs.

## Materials and methods

### Participants

The participants of this study were 10 subjects with right hemisphere cortical damage (**RH**, Male = 4, Female = 6) and 15 healthy controls (**HC**, Male = 5, Female = 10). Both groups were matched in age (**RH**: *M* = 61.9, *SD* = 11.9; **HC**: *M* = 62.80, *SD* = 4.12) and education (**RH**: *M* = 13.63, *SD* = 1.36; **HC**: *M* = 13.93, *SD* = 1.75). The average time since injury in the ABI group was 55.8 months (*SD* = 34.84; *MIN* = 13, *MAX* = 114). Participants were referred by neurologists from Bangor University and the North Wales Brain Injury Service. The study was approved by the School of Psychology's Ethics Committee and by the North Wales Research Ethics Committee. Exclusion criteria for the neurological group were having a non-focal lesion, duration of less than 6 months since the brain injury, and moderate to severe language impairment. The details of patient's lesion location are described in Table 1.

**Table 1:** **Clinical details of participants with acquired brain injury**.

**Age/Sex**	**Months since injury**	**Location**	**Etiology**
57 F	84	Right prefrontal	MCA stroke
50 M	20	Right prefrontal	MCA ACA stroke
73 F	88	Right prefrontal	MCA stroke
45 M	70	Right prefrontal	ACoA SAH
74 M	20	Right ventro-lateral prefrontal cortex, basal ganglia	MCA stroke
65 M	65	Right frontal and TPJ	MCA stroke
46 F	114	Right prefrontal	MCA stroke
63 F	60	Right prefrontal and TPJ	MCA stroke
78 F	13	Right prefrontal	MCA stroke
68 F	24	Right fronto-parietal	MCA stroke

ACoA, anterior communicating artery aneurism; SAH, subarachnoid hemorrhage, MCA, middle cerebral artery; ACA, Anterior cerebral artery; TPJ, Tempo Parietal Junction.

### Procedure

The assessment took place mainly at Bangor University. In cases where participants with brain injury were not able to travel, researchers tested them at home. The assessment involved two sessions, of 2 h each. As a main introduction of the research, they were told that they would be asked to recall personal events in relation to certain emotions. In addition, they would have to watch some movies on the computer, and report on how they made them feel. For a detailed description of the procedure adapted for this study see Salas et al. (2012). During the first session, participants completed a set of measures of overall cognitive functions and the Film Clip task. The Affective Story Recall was administered during the second session, along with other neuropsychological tasks that are not described here.

### Mood induction procedures

#### External mood induction procedure: film clips

In this task participants are asked to watch a series of film clips and report their emotional experience while watching the clips. For that purpose, participants were placed in front of a 15″ laptop screen. Headphones were provided to avoid any possible distracting noise. The following instruction was offered: “I will ask you to watch a couple of short film clips. Please pay attention and watch them carefully”. After each film clip a self-report questionnaire was administered. Before each “emotional” video a neutral video was presented, and the following instruction was given: “Now I would like you to watch this video and try to relax and clear your mind of any thoughts”. The “emotional” clips [amusement-sadness] were counterbalanced across participants of each group to avoid order effects.

In relation to the neutral and emotional clips used, all of them have been previously validated regarding their capacity to elicit specific and discrete emotions (Gross and Levenson, 1995; Rottenberg et al., 2007). The neutral clips, *Sticks* (Gross and Levenson, 1995), were 60s in length and showed abstract shapes and colors. The amusement clip, *Bill Cosby Himself* (Cosby, 1983) showed a stand-up comedy performance by Bill Cosby (121 s). The sadness clip, *The Champ* (Zeffirelli, 1979), showed a young boy facing the sudden death of his father after a boxing match (171 s).

#### Internal mood induction procedure: affective story recall

The Affective Story Recall task (Turnbull et al., 2004) was used as an internal MIP for both amusement and sadness. In this task participants are asked to recall personal events from their lives related to specific emotions. As with the film clips, two neutral recall conditions (going shopping and fixing a meal) are used before each emotional recall as baselines. Each recall was prompted with the following phrase: “Try to recall an event in your life that has caused you to feel… (e.g., amusement). Try to be very detailed about the way you feel.” Each participant had a maximum of 3 min to describe the event, although they could use more time if they felt they have not finished yet the story after 3 min. A minimum of time was not set, in order to avoid the possibility that task instructions could activate negative emotions by adding pressure and anxiety to the recollection. The same self-report questionnaire, administered after each clip, was also administered after each recollection.

### Measures

#### Neuropsychological assessment

A brief battery of neuropsychological tasks was used to obtain a profile of the participants' cognitive functioning. The Mini Mental State Examination (*MMSE*; Rovner and Folstein, 1987) was employed as a basic screening tool for overall cognition. Sustained attention and divided attention were measured using the *Telephone Search* from the Test of Everyday Attention (*TEA*, Robertson et al., 1994). Comprehensive language was assessed with the Token Test (De Renzi and Faglioni, 1978). The Bells Cancelation test (Gauthier et al., 1989) and the Rey-Osterrieth Figure Copy (Stern et al., 1994) were employed to explore visuo-spatial abilities. In order to obtain a profile of verbal and visual memory capacities, the Logic Memory task (*WMS-R*, Wechsler, 1987) and the Rey-Osterrieth Figure recall (Stern et al., 1994) were used, respectively. The Frontal Assessment Battery (*FAB*, Dubois et al., 2000) was used as a general measure of executive function abilities. Nevertheless, more specific tasks were also considered to explore such functions in detail. Working memory was assessed using Digits, and abstraction ability was explored using Similarities, both from the WAIS-III (Wechsler, 1997). Finally, verbal fluency from the D-KEFS (Delis et al., 2001) was used to obtain a measure of letter fluency, category fluency and category switching.

#### Emotional assessment

In order to capture the emotional experience of participants, after each clip and affective story, a self-report measure was adapted from the PANAS-X (Watson and Clark, 1994). For a detailed description of the construction of this scale see Salas et al. (2013). The PANAS-X is a 60-item self-report that assesses specific emotional states. It has a general dimension of positive and negative affect, but also includes specific scales for discrete emotions. Participants are asked to rate each of the 60 emotional words (e.g., cheerful, hostile, shaky) indicating the extent to which they felt each emotion according to a 5-point scale (very slightly or not at all, a little, moderately, quite a bit, and extremely). For the purpose of this study, following Salas et al. (2012) a total of 20 emotional words were selected from the discrete emotion scales, with 5 emotional words for each of the four basic emotions [*Joy*: cheerful, delighted, happy, joyful, energetic; *Sad*: downhearted, sad, blue, lonely, alone; *Anger*: disgusted, angry, loathed, irritable, hostile; *Fear*: shaky, afraid, nervous, scared, frightened].

## Data analysis

The average score of each PANAS' subscale [Fear, Sadness, Joy, and Anger] was calculated, for each eliciting stimulus [Neutral 1, Sadness, Neutral 2, Joy] on both tasks [ASR and FC]. To test the Selectivity and Intensity hypothesis, the PANAS score for each stimulus was compared using a mixed-anova test. More specifically, to answer the Selectivity hypothesis, the analysis was conducted separately for each stimulus and tasks, with the four subscales of the PANAS treated as the within-subject variables and controls vs. patients as the between-subject variable. The differences between the target emotion score and the other emotions were calculated using a simple contrast, with the target emotion as reference. In relation to the Intensity hypothesis, separated analyses for each stimulus were conducted, using the target emotion in both tasks [e.g., sadness or joy] as the within-subject variable, and controls vs. patients as the between-subject variable. In both analyses, when the sphericity assumption was not respected, Greenhouse-Geisser correction was used (for Greenhouse-Geisser's between 0.9 and 0.7), or multivariate Pillai's Trace statistic was reported (for Greenhouse-Geisser's less than 0.7). Finally, the exploration of the interaction term in the mixed models allowed to test the third hypothesis (Right Hemisphere), and assess whether the comparisons in the within model differed between groups.

## Results

### Neuropsychological assessment

Data offered by the neuropsychological assessment is consistent with existing literature on attentional and executive impairment after right hemisphere damage (see Table 2). Individuals with RH lesions exhibited scores above the cut-off point on the MMSE, suggesting preserved overall cognition. However, their performance on this screening task was significantly lower than controls. When assessed in more detail, several cognitive areas appeared as preserved: divided attention [Telephone Search TEA], language [Token Test] and memory [Logical Memory and Rey-Osterrieth recall].

**Table 2:** **Summary of Neuropsychological Assessment**.

**Cognitive function**	**Task**	**RH (**N** = **10**)**		**HC (**N** = **15**)**		***p***
		***M***	***SD***	***M***	***SD***	
*Overall cognition*	Mini Mental State Examination	27.8^*^	1.68	29.00	1.25	0.02
*Attention*	Sustained attention [Telephone Search TEA]	7.88^**^	1.64	11.00	1.56	0.001
	Divided attention [Telephone Search TEA]	10.00	4.89	11.00	1.79	0.24
*Language*	Token test	30.40	1.43	31.13	1.35	0.10
*Visuo-spatial abilities*	Bell test	21.6^**^	4.67	27.87	4.58	0.001
	Rey-Osterrieth copy	30.10	6.59	34.00	3.12	0.06
*Memory*	Logical Memory Immediate recall [WMS R]	12.50	3.26	13.67	3.43	0.20
	Logical Memory Delayed recall [WMS R]	14.30	3.65	16.33	3.77	0.10
	Logical Memory Recognition [WMS R]	13.22	1.39	13.86	1.52	0.16
	Rey-Osterrieth recall	10.89	5.12	14.40	5.60	0.68
*Executive functions*	Working memory [Digits WAIS III]	8.90	2.60	10.77	2.24	0.39
	Abstraction [Similarities WAIS III]	9.63	3.29	11.86	3.31	0.65
	Letter fluency [D-KEFS]	7.88^*^	2.22	10.71	3.26	0.02
	Category fluency [D-KEFS]	7.75^**^	1.90	11.07	2.12	0.001
	Category switching [D-KEFS]	8.25^**^	3.15	12.43	2.17	0.001
	Frontal Assessment Battery Total Score	14.9^*^	1.91	16.62	1.93	0.02

* p < 0.05;

** p < 0.001.

The RH group presented with impaired performance on visual scanning [Bell Cancelation tasks] and sustained attention [Telephone Search]. These findings are consistent with evidence suggesting that visual scanning impairment is common after RH lesions (Corbetta and Shulman, 2011) and that sustained attention is often compromised after damage to this hemisphere (Robertson et al., 1995, 1997; Leclercq et al., 2002), particularly when the prefrontal region is involved (Wilkins et al., 1987; Rueckert and Grafman, 1996; Stuss et al., 2002; Picton et al., 2006; Shallice et al., 2008; Molenberghs et al., 2009).

In relation to executive functions, patients with RH damage presented a mixed profile. Their overall score on the FAB, a screening for executive function abilities, was above the proposed cut-off score [12], however, their performance was significantly lower compared to healthy controls. Working memory [digits] and abstraction [similarities] were within the normal range. Nevertheless, measures of verbal fluency and task switching [letter fluency, category fluency and category switching, D-KEFS] appeared impaired relative to population norms, and were significantly lower when compared to controls.

### Emotion elicitation

The present study tested three hypotheses: (a) Selectivity: that internal and external MIPs will elicit the target emotion with grater intensity than the non-target emotions, this in both groups; (b) Intensity: that target emotions elicited by the internal MIP will show greater intensity than the ones generated by the external MIP, in both groups; (c) Right Hemisphere: that the neurological group would present lower levels of negative emotional intensity compared to the healthy control group when exposed to internal or external stimuli.

#### Selectivity

**Figure 1:**
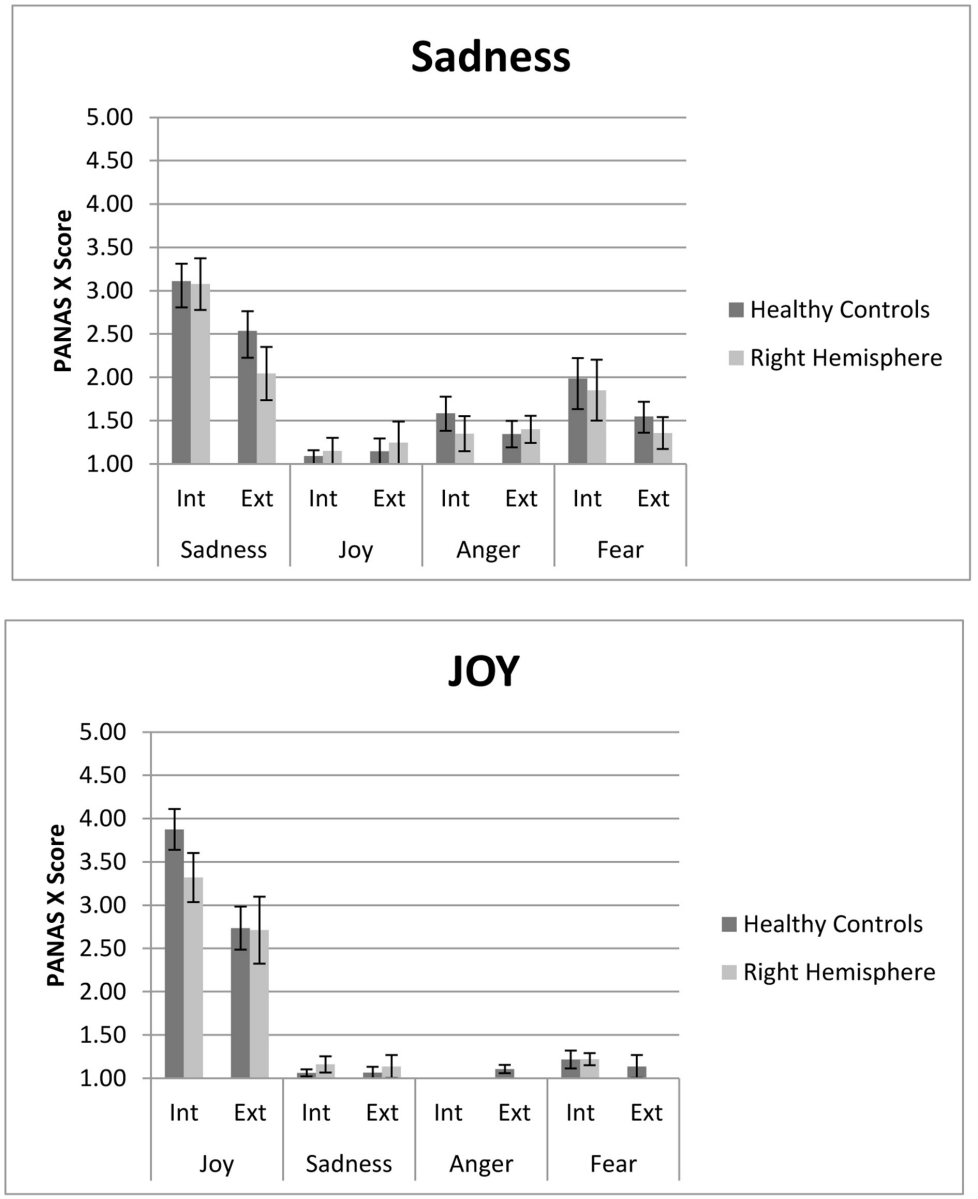
**PANAS score for internal (ASR) and external (FC) Sad and Joy stimuli**. Both right hemisphere participants and healthy are represented. In each the internal and external procedure induce slectively the target emotion compared to non-target emotions. However, the internal procedure generates higher levels of experienced emotion. Participants with right hemisphere damage do not differ from controls in both measures.

Both MIPs elicited the two target emotions (e.g., sadness and joy) effectively, with higher levels of intensity than the other emotions considered in the PANAS (e.g., sadness/joy/anger/fear) (See Figure 1). This result demonstrates that both internal and external MIPs are effective in inducing an emotion selectively, for people with and without brain injury (see Table 3).

**Table 3:** **PANAS score differences between target and non-target emotions for the internal (ASR) and external (FC) MIP**.

**Mood induction procedure**	**Target emotion**	**Non-Target emotion**	**Paired differences**	***df***	***F***	***p***	**ηρ^2^**	***r***
*Internal*	*Sad*	Joy	9.91	1.19	89.35	<0.001	0.83	0.91
*(Affective story recall)*		Anger	8.00	1.19	60.10	<0.001	0.76	0.87
		Fear	5.81	1.19	36.44	<0.001	0.66	0.81
	*Joy*	Sad	12.65	1.21	154.80	<0.001	0.88	0.94
		Anger	13.17	1.21	202.65	<0.001	0.91	0.95
		Fear	12.08	1.21	155.10	<0.001	0.88	0.94
*External*	*Sad*	Joy	5.83	1.22	25.64	<0.001	0.54	0.73
*(Film clips)*		Anger	4.92	1.22	20.48	<0.001	0.48	0.69
		Fear	4.37	1.22	17.76	<0.001	0.45	0.67
	*Joy*	Sad	8.17	1.22	41.74	<0.001	0.66	0.81
		Anger	8.3	1.22	54.03	<0.001	0.71	0.84
		Fear	8.21	1.22	45.70	<0.001	0.68	0.82

Differences in the average intensity between target and non-target emotions, for the two emotional stimulus, were observed in the internal [Sadness: *F*_(3, 57)_ = 34.34, *p* < 0.001, ηρ^2^ = 0.64, *r* = 0.80; Joy: *v* = 0.92, *F*_(3, 19)_ = 74.36, *p* < 0.001, ηρ^2^ = 0.92, *r* = 0.96] and external [Sadness: *F*_(2.44, 53.67)_ = 14.71, *p* < 0.001, ηρ^2^ = 0.40, *r* = 0.63; Joy: *v* = 0.75, *F*_(3, 20)_ = 19.45, *p* < 0.001, ηρ^2^ = 0.75, *r* = 0.87] MIP. All the planned comparisons between target and other emotions were significant in *both* MIPs, as seen in Table 2. No differences across groups were found [Interaction emotion*group; Sadness Internal: *F*_(3, 57)_ = 0.19, *p* = 0.91, ηρ^2^ = 0.01, *r* = 0.10; Joy Internal: *v* = 0.12, *F*_(3, 19)_ = 0.84, *p* = 0.49, ηρ^2^ = 0.12, *r* = 0.34; Sadness external: *F*_(2.44,53.67)_ = 1.13, *p* = 0.34, ηρ^2^ = 0.05, *r* = 0.22; Joy external: Joy: *v* = 0.14, *F*_(3, 20)_ = 1.07, *p* = 0.38, ηρ^2^ = 0.14, *r* = 0.37]. Thus, the comparison between the target emotion and the other three emotions was statistically significant for each stimulus, in both tasks, showing the same pattern in healthy controls and neurological group.

#### Intensity

The internal MIP elicited higher levels of intensity in the target emotion when compared to the external MIP, for both sadness and joy. No significant differences were found between groups. This result suggests that internal MIPs are more effective than external MIPs in triggering intense emotional experience (see Table 4).

**Table 4:** **Comparison of the target emotion's level of intensity when using an internal (ASR) or external (FC) MIP**.

**Target emotion**	**Internal (ASR)**		**External (FC)**		**Paired differences**					
	**Mean**	***SD***	**Mean**	***SD***	**ASR-FC**	***df***	***F***	***p***	**ηρ^2^**	***r***
Sadness in SAD	15.50	3.90	12.40	4.30	3.10	1.18	22.53	<0.001	0.56	0.75
Joy in JOY	18.05	4.53	13.18	5.09	4.87	1.20	11.1	<0.01	0.36	0.60

The internal MIP generates significantly higher levels of intensity.

The average intensity of the target emotion in the internal MIP was higher than the external MIP for both sad [*F*_(1, 18)_ = 22.53, *p* < 0.001, ηρ^2^ = 0.56, *r* = 0.75] and joy [*F*_(1, 20)_ = 11.10, *p* = 0.003, ηρ^2^ = 0.36, *r* = 0.60] stimulus. Thus, the comparison between internally and externally generated target emotions was statistically significant for each stimulus, with no differences between participants with and without brain damage [Sadness: *F*_(1, 18)_ = 0.99, *p* = 0.33, ηρ^2^ = 0.05, *r* = 0.22; Joy: *F*_(1, 20)_ = 2.08, *p* = 0.17, ηρ^2^ = 0.09, *r* = 0.30].

It is interesting to note, at an individual subject level, that 10/10 of the participants with RH cortical damage reported *some* level of positive and negative emotion during the affective story recall [Joy Range: 1.8–4.4; Sadness Range: 1.8–4.2]. A similar profile was observed in controls [Joy Range: 2.4–5; Sadness Range: 2–4.8]. In relation to the film clips, only two participants reported not experiencing any degree of emotional response, one of them to the sad clip, and the other to the joy clip. In general, participants with RH cortical damage [Joy Range: 1.2–3.6; Sad Range: 1–3.4] and controls showed a similar range of performance [Joy Range: 1.2–4; Sad Range: 1.2–4.4].

#### Right hemisphere

As reported above, participants with unilateral RH cortical damage did not differ from healthy controls in level of intensity reached by positive and negative target emotions, using internal and external MIPs. No significant interactions were observed between group and levels of intensity, for both positive and negative emotions. In conclusion, the data did not support the hypothesis suggesting that RH patients would not have selectively lower levels of emotional experience. Results for all three hypotheses are described in Figure 1.

## Discussion

The goal of this study was twofold. Firstly, it explored the effectiveness of internal and external MIPs in an older adult sample of people with RH cortical damage, and healthy controls. Secondly, it tested whether participants with RH cortical lesions were able to experience negative emotions using an internal and external MIPs.

The first finding of this study is that the levels of intensity were higher for the *internally* generated material. This is a replication of the results reported by Salas et al. in a student sample (2012). The present study extends these results by suggesting that a similar pattern is also reported in elderly people, and also in those with acquired brain injury. Interestingly, the material recalled by participants (see Appendix) was qualitatively similar for patients and controls and reflected the quantitative scores generated in the PANAS-X. In addition, it was also found that levels of emotional intensity, measured by self-report, were not significantly different between participants with RH unilateral damage and healthy controls, for both positive [joy] and negative [sadness] emotions.

The results from this study contribute to a growing literature on the elicitation of emotion (Coan and Allen, 2007). When these results are interpreted in relation to previous studies comparing the efficacy of internal and external MIPs (Salas et al., 2012), they appear to suggest that the higher levels of emotion generated by the *internal* MIP are independent of age, for this phenomenon is observed in both young and elderly adult populations. This is an interesting finding for the literature on emotion, which has tended to focus on the *structure* of emotional intensity (e.g., Sonnemans and Frijda, 1994; Verduyn et al., 2009), and the individual determinants of intensity levels (e.g., Sonnemans and Frijda, 1995; Neumann et al., 2001; Lynch et al., 2001), with less consideration of the impact of the MIPs used (for a review see Salas et al., 2012). This study offers important evidence supporting the view that emotion can be more intensely triggered when using personally relevant material.

The data obtained by this study are also of relevance to the elicitation and assessment of emotion in people with acquired brain damage (Levenson, 2007; Levenson et al., 2008). It shows that, in the same way as healthy controls, people with right hemisphere lesions also generate higher levels of positive and negative emotion when using *internal* MIPs. This finding suggests that the recollection of personal affective memories is also a powerful elicitor of emotional states in this population, and that such recollection does not appear to be compromised by the cognitive difficulties commonly associated with RH lesions [sustained attention and executive impairment]. In fact, an exploratory analysis of correlations between the level of emotional experience [self-report scores] and cognitive abilities [sustained attention, working memory, delayed verbal recall, abstraction, verbal fluency and divided attention] did not show any clear pattern of association^1^.

In terms of the RH debate on emotional experience, these data suggest that the capacity to experience positive and negative emotions, when measured by self-report, is preserved in patients with RH cortical lesions, offering group study support for previous case studies (Turnbull et al., 2004; Tondowski et al., 2007). Taken together, these findings also appear to challenge the valence hypothesis, which proposes that the experience of negative emotions is compromised after right cortical damage (Davidson, 1992a,b; Davidson and Irwin, 1999; Davidson, 2001; Craig, 2005).

In addition, these data offers supporting evidence for a newly developing perspective in affective neuroscience, which suggest that emotion generation depends heavily on *deep* subcortical structures, such as the brainstem [e.g., periacqueductal gray] (Panksepp, 1998, 2011; Walla and Panksepp, 2013), and that the neocortex would have a role not in emotion generation, but in the re-presentation and cognitive regulation of affects and feelings (Panksepp, 1998, 2011; Damasio et al., 2000; Holstege et al., 2003; Ochsner and Gross, 2007; Solms and Panksepp, 2012; Salas et al., 2013, 2014; Walla and Panksepp, 2013; Turnbull et al., 2014a,b).

In relation to the role of the cortex in emotion generation, for example, it has long been reported that emotional experience is preserved after cortical damage and cortical atrophy (Shewmon et al., 1999; Merker, 2007). In a recent single case study, Damasio et al. (2012) reported findings from a man [patient B] with extensive bilateral damage to the insula, a cortical area often suggested (Craig, 2008, 2009, 2010) by neuroimaging studies as involved in bodily feelings and the experience of emotions. The authors found that all aspects of feelings appeared to be intact in patient B, suggesting that the insula cannot have an *exclusive* role in the processing of feelings. Taken together with the findings reporting that cortical areas are not involved in emotional experience [as reviewed above], these data suggest that the experience of emotion is mediated by subcortical [basal ganglia] and specially deep subcortical [brain stem complex] brain areas (Panksepp, 1998, 2011; Damasio, 2000).

It is also clear that many subcortical areas often associated with emotion are not core to emotional *experience*. For example, one study that compared individuals with amygdala damage and controls, found no differences between groups in terms of dispositional affect and day-to-day generation of affective states (Anderson and Phelps, 2002). In a case study of a subject with bilateral amygdala damage [SM] (Feinstein et al., 2011), it was found that only the induction of the experience of fear appeared to be impaired, and that the triggering and experience of other emotions was spared. Furthermore, in a subsequent case group study, Feinstein et al. (2013) explored the experience of fear and panic in individuals with bilateral amygdala damage, using a novel paradigm that involved CO_2_ inhalation. Interestingly, it was reported that all three patients experienced panic attacks, and that the bilateral amygdala lesions did not interfere with the capacity to express or experience fear, or fear related emotions [panic or anxiety]. The authors offer several possible interpretations for these challenging data. They suggest, for example, that the amygdala may have a regulatory role in the inhibition of fear. They also proposed that previous studies reporting the absence of fear experience in patients with amygdala lesions have used exteroceptive stimuli [processed cortically via visual and auditory pathways], while CO_2_ appears to engage interoceptive afferent sensory pathways, that project to the diencephalon, insular cortex, and deep subcortical structures such as the brainstem.

The present study has two main limitations. The first might be argued to be small sample size, though we note that previous studies (Turnbull et al., 2004; Tondowski et al., 2007) have been at the level of single case reports. Nevertheless, these data would clearly benefit from confirmation with a larger population. The second limitation is that this study only focuses on subjective emotional experience, not capturing physiological or behavioral correlates. This is an important issue, since there is evidence pointing to relatively modest levels of convergence between subjective, behavioral and physiological findings, together with reports of dissociations between these response systems (for a review see Mauss and Robinson, 2009). These data has led some authors to recommend the use of multiple measures that can capture different aspects of the emotional response (Mauss et al., 2005; Lyons et al., 2013; Walla et al., 2013), while others have suggested efforts to improve the way self-reports are used, in order to develop better first person methods (Nielsen and Kaszniak, 2007). However, thus far the literature on emotional changes after brain damage has by and large failed to consider the subjective *experience* of emotions, mostly focusing on individuals' ability to *perceive* or *express* feelings [see introduction]. A main goal of this preliminary study has been to correct this bias.

It is important to note that the effort to investigate emotional experience does not only have value for the neuroscientific community, but is also important for those interested in the link between neuroscience and psychoanalysis. The main goal of the neuropsychoanalytic enterprise is to understand the neural basis of complex psychological processes that lie at the heart of what we call subjective experience, of which emotion is a core element (Solms and Turnbull, 2011; Turnbull and Solms, 2004, 2007). In this context, the observation of emotional changes, using a first person perspective has been a key methodological tool (Kaplan-Solms and Solms, 2000; Salas et al., 2014). This study has attempted to follow this tradition, by putting back, as a focus of research, subjective experience while retaining a degree of experimental control.

It is interesting to note that our findings challenge previous data from behavioral [facial] (e.g., Borod et al., 2002) and physiological studies (Heilman et al., 1978; Morrow et al., 1981), which suggest that individuals with RH damage are impaired in expressing, understanding and physiologically reacting to emotional stimuli. There are a number of possible explanations for this apparent inconsistency. One explanation might be the reliable existence of dissociations between the subjective, cognitive and somatic/behavioral components of the emotional response - we currently have incomplete data to make this judgment. The design of future studies that consider all three components at once (Mauss et al., 2005) will offer valuable insight. An alternative explanation might be that perceptual/expressive and physiological deficits, after RH damage, are not a direct consequence of impaired *emotional* reactivity, but the result of deficits in cognitive abilities that are necessary to trigger adequate emotional responses. Thus, the patient might lack the cognitive abilities [e.g., attention, high level visuo–spatial skills] to distinguish (say) a sad face, but retains the experience of sadness when elicited by other routes. A final explanation might be that the low emotional reactivity reported by early studies is a consequence of dynamic psychological factors, of a “defensive” nature, perhaps related to impairment of emotion *regulation* rather than in the direct experience of emotion (Turnbull et al., 2014a).

For decades emotional changes after brain injury have contributed to our understanding of the neural basis of emotional processes (Damasio, 1994; Borod, 2000; Gainotti, 2001; Robinson, 2006; Adolphs, 2007; Rosen and Levenson, 2009; Feinstein, 2013). However, little attention has been placed on the methods used to *trigger* emotional responses (Levenson, 2007; Levenson et al., 2008). This study has contributed to the field by showing that internal and external forms of elicitation are both effective in triggering selective emotional states, although the internal procedure generates higher levels of intensity. More importantly, it suggests that these methods are also effective in triggering negative emotional states in patients with RH unilateral damage, a population that has been traditionally described as impaired in the capacity to experience negative emotion.

### Conflict of interest statement

The authors declare that the research was conducted in the absence of any commercial or financial relationships that could be construed as a potential conflict of interest.

## Footnotes

^1^ The only significant correlations found in the exploratory analysis were: ASR [Joy]^*^Sustained Attention [*r* = 0.45, *p* < 0.05]; Film Clip [Sad]^*^Category Fluency [*r* = 0.55, *p* < 0.01]; Film Clip [Joy]^*^Abstraction [*r* = 0.44, *p* < 0.05].
