# Supplementary material for: Internally and externally generated emotions in people with acquired brain injury: preservation of emotional experience after right hemisphere lesions
Source: Front Psychol. 2015 Feb 16;6:101. doi: 10.3389/fpsyg.2015.00101 (PMC4329804; doi:10.3389/fpsyg.2015.00101)
Supplement: Supplementary file 1 [file DataSheet1.PDF]

IDENTIFICATION	SAD	PANAS X	JOY	PANAS
Male, 63<br>*Right MCA*	We had a daughter who died…cot death. She was sleeping in the cot, and my wife found her just lying there, dead (5s). She came running out to find me and I tried to do CPR on her. It is very hard to do that on a four month old baby. Because you don't know how to breathe and how much to press.	SAD: 3.8<br>JOY: 1.0<br>FEAR: 3.0<br>ANGER: 1.0	Getting married… I felt very happy. I was a little bit frightened as to what would happen next (13s) [*prompt by examiner*: could you describe the event?]. I felt very happy that time. My in-laws, my wife's parents were able to arrange the wedding, the reception and the party afterwards (15s). [examiner: is that it?]. Yes.	JOY: 2.4<br>SAD: 1.0<br>FEAR: 1.4<br>ANGER: 1.0
Female, 57<br>*Right MCA*	When my son died. We lived next door when it happened. And my son was living in Brighton with his partner. It was a Sunday morning and my husband was at the supermarket, as every Sunday morning. My daughter went to her friends and I got a phone call about ten o'clock in the morning. It was a girl on the phone and she said 'are you John's mother?' and I said yes. And she said 'I'm just phoning to say he is dead'. And that was it. And I phoned my brother, for my brother to go and get my daughter and my husband. We phoned the police because we did not know if it was true, but the police didn't come to us until about half past seven at night, when they came to confirm that it was John. He died of a heart attack. I don't know how it happened or why. We have no idea, he had never been ill before.	SAD: 3.8<br>JOY: 1.0<br>FEAR: 2.2<br>ANGER: 1.0	When we renewed our wedding vows with my husband. We have been married for twenty five years. We had been to a wedding like twelve months before, to my husband's niece, who got married in a little church in Anglesey. And after the wedding we went to the Beaumaris hotel for dinner and a disco. It was a nice day and everything went well. So my husband decided that we had to renew our wedding vows, because when we got married…we got married in London… in the Registery Office, and we got no family there at all. So we did the same thing in the little church. My husband thought that would be a good idea. So we went there with some friends and family and then to the Beaumaris Hotel. We had a set meal for everybody, and then we had a disco, so we had to stay there all day. That was it.	JOY: 2.3<br>SAD: 1.0<br>FEAR: 1.0<br>ANGER: 1.0
Male, 67<br>*Healthy Control*	That is easy. When my grandfather died. Because I didn't know my father. He was killed in world war II. He was a part of the Cheshire battalion. So practically my grandfather was my father during my whole life. And he died at quite an early age from heart problems. And I was quite young at the time, and I remember being rather overwhelmed with sadness, and even though he had been ill. It was just one day. He was ill and suddenly he was gone. It was a pity really.	SAD: 3.8<br>JOY: 1.2<br>FEAR: 1.2<br>ANGER: 1.0	Well there have been plenty of happy times, fortunately. But one that I recall…I have always been interested in underground exploration and government bunkers and that sort of things. And I remember the first time I went to a place called Mountain Farley. It was huge. I went down the sloping shaft and there were tunnels everywhere and they were on for miles and miles and miles and miles. And it had all sort of interesting artefacts, fans, and motors and stuff. And I have never seen a piece of engineering quite like it in my life. So that was a happy couple of days exploring. Very satisfying.	JOY: 4.6<br>SAD: 1.0<br>FEAR: 1.0<br>ANGER: 1.0

Female, 59<br>*Healthy Control*	It was a couple of years ago. We were planning a holiday in America. My cousin was fully planned. We were going to go to Las Vegas. And he said that his wife was away for tests And he said that he would be back to us once he had the results. And he found that apparently it was very bad news. The doctors gave her about six weeks. They found cancer, obviously. So I asked him if he wanted me to come to visit, and he said yes. So we made some arrangements. So we flew out there, and my husband managed to sort out several things we needed to do before. And we arrived there on the Saturday. She wasn't too bad to start with. She kind of resigned herself to it. She was quite amazing really, she was so brave. She was actually joking about being able to eat whatever she wanted, after years of watching her weight. It was very difficult at the beginning to believe that there was anything wrong. But on a Tuesday the cancer must have gone to her brain and I thing that she had a slight stroke. A daughter was taking care of her as a nurse, she did not want to go to a hospital. She died that Friday. So that was a very very sad time. But I'm glad that I was there.	SAD: 3.0<br>JOY: 1.0<br>FEAR: 1.2<br>ANGER: 1.8	I suppose I have to think years back, when we went to America and had these wonderful holidays in a motor-home. We sold the house and bought a motor-home. So we travelled quite a lot and we got to see many places that are off the tracks…so they were very happy times. We went down to Mexico, been down to Arizona, Texas, and Louisiana... oh well... they were really happy times.	JOY: 4.6<br>SAD: 1.0<br>FEAR: 1.6<br>ANGER: 1.0

Appendix. Vignettes from the Internally Generated Mood Induction Procedure (ASR). Sad and Joy stories are transcribed for two participants with Right Hemisphere damage and two Healthy Controls. The self-report scores from the PANAS on each story are also described.
